# Effects of glycyrrhizin on the pharmacokinetics of paeoniflorin in rats and its potential mechanism

**DOI:** 10.1080/13880209.2019.1651876

**Published:** 2019-08-20

**Authors:** Hongjuan Sun, Jingfeng Wang, Juan Lv

**Affiliations:** a Department of Pediatrics, Liaocheng Dongchangfu People’s Hospital, Liaocheng, China;; b Department of Pharmacy, Yantai Affiliated Hospital of Binzhou Medical University, Yantai, China

**Keywords:** P-gp, CYP450, drug–drug interaction

## Abstract

**Context:** Paeoniflorin is reported to possess numerous pharmacological activities. Paeoniflorin and glycyrrhizin are always used together for the treatment of disease in China clinics; however, the drug–drug interaction between glycyrrhizin and paeoniflorin is still unknown.

**Objective:** This study investigates the effects of glycyrrhizin on the pharmacokinetics of paeoniflorin in rats.

**Materials and methods:** The pharmacokinetics of orally administered paeoniflorin (20 mg/kg) with or without glycyrrhizin pre-treatment (at a dose of 100 mg/kg/day for 7 days) were investigated in male Sprague–Dawley rats using LC-MS/MS. Additionally, Caco-2 cell transwell model and rat liver microsome incubation experiments were also conducted to investigate its potential mechanism.

**Results:** The results showed that when the rats were pre-treated with glycyrrhizin, the *C_max_* of paeoniflorin decreased from 59.57 ± 10.24 to 45.36 ± 8.61 ng/mL, and *AUC_0-inf_* also decreased from 282.02 ± 35.06 to 202.29 ± 28.28 μg·h/L. The *t_1/2_* value of paeoniflorin decreased from 8.48 ± 2.01 to 5.88 ± 1.15 h (*p* < 0.05). The Caco-2 cell transwell experiments indicated that glycyrrhizin could increase the efflux ratio of paeoniflorin from 2.71 to 3.52, and the rat liver microsome incubation experiments showed that glycyrrhizin could significantly increase its intrinsic clearance rate from 53.7 ± 4.6 to 85.6 ± 7.1 μL/min/mg protein.

**Conclusions:** These results indicated that glycyrrhizin could affect the pharmacokinetics of paeoniflorin, and it might work through decreasing the absorption of paeoniflorin by inducing the activity of *P-gp* or through increasing the clearance rate in rat liver by inducing the activity of CYP450 enzyme.

## Introduction

Paeoniflorin, a monoterpene glycoside, is one of the principal bioactive components extracted from the root of *Paeonia lactiflora* Pall. (Paeoniaceae) that has been used in Traditional Chinese Medicine for thousands of years (Shao et al. 2016; Xu et al. 2018). Paeoniflorin possesses numerous pharmacological activities, such as antioxidative, anti-inflammatory and hepatoprotective effects (He and Dai 2011). Because of its safety and immunoregulatory effects, paeoniflorin has been widely used in treating various inflammatory and autoimmune diseases, such as systemic lupus erythematosus, rheumatoid arthritis, Sjogren’s syndrome, allergic contact dermatitis and psoriasis (Zhang and Dai 2012; Zhao M et al. 2012; Shi et al. 2015; Zhang et al. 2016; Zhao J et al. 2016).

Liquorice is the root of *Glycyrrhiza uralensis* Fisch. or *Glycyrrhiza glabra* L., (Leguminosae) (Akao et al. 1994; Asl and Hosseinzadeh 2008). Liquorice has been commonly used together with other herbs to enhance the effects of other ingredients or to reduce toxicity in traditional Chinese medicine (Chen et al. 2009; Sun et al. 2013; Han et al. 2014; Bhattacharjee et al. 2015). It has been reported that the glycyrrhizin, a triterpenoid saponin that isolated from liquorice, has anti-inflammatory, hepato-protective and antitumour properties. Some research articles have indicated that the drug–drug interaction might occur when they are co-administered (Hu et al. 2003; Gao QT et al. 2004; Chen et al. 2012; Tai et al. 2014; Feng et al. 2015; Gao X et al. 2016). To the best of our knowledge, there are little data available for the effects of glycyrrhizin on the pharmacokinetics of paeoniflorin. As Chinese medicines have become more and more popular all over the world due to their natural origin, glycyrrhizin and paeoniflorin are always co-administered in clinical for the treatment of different diseases, and therefore, the drug–drug interaction between glycyrrhizin and paeoniflorin should be investigated.

This study investigates the effects of glycyrrhizin on the pharmacokinetics of paeoniflorin in rats. The *in vivo* pharmacokinetics of paeoniflorin in rats with or without pre-treatment with glycyrrhizin were determined using LC-MS/MS method. Additionally, to investigate its potential mechanism, the effects of glycyrrhizin on the transport of paeoniflorin were investigated in the Caco-2 cell transwell model, and the effects of glycyrrhizin on the metabolic stability of paeoniflorin were determined using rat liver microsome incubation experiments.

## Materials and methods

### Chemicals and reagents

Paeoniflorin (purity >98%) and hyperoside (purity >98%) were purchased from the National Institute for the Control of Pharmaceutical and Biological Products (Beijing, China). β-nicotinamide adenine dinucleotide phosphate (NADP^+^) and Lucifer yellow were provided by Sigma (St. Louis, MO, USA). Rat liver microsomes were purchased from BD (Woburn, MA, USA). Acetonitrile and methanol were purchased from Fisher Scientific (Fair Lawn, NJ, USA). Ultrapure water was prepared with a Milli-Q water purification system (Millipore, Billerica, MA, USA). All other chemicals were of analytical grade or better.

### Animal experiments

Male Sprague–Dawley rats weighing 230–250 g were provided by the experimental animal centre of the Liaocheng Dongchangfu People’s Hospital (Shandong, China). Rats were bred in a breeding room at 25 °C with 60 ± 5% humidity and a 12 h dark–light cycle. Tap water and normal chow were given *ad libitum*. All of the experimental animals were housed under the above conditions for a three-day acclimation period and fasted overnight before the experiments. The experiments were approved by the Animal Ethics Committee of Liaocheng Dongchangfu People’s Hospital.

### *In vivo* pharmacokinetic study

To evaluate the effects of glycyrrhizin on the pharmacokinetics of paeoniflorin, the SD rats were divided into two groups of six animals each. The test group was pre-treated with glycyrrhizin by intragastric gavage at a dose of 100 mg/kg/day (dissolved directly in normal saline at a concentration of 20 mg/mL) for 7 days before the administration of paeoniflorin (Guo et al. 2018). Then paeoniflorin (dissolved in normal saline containing 0.5% methylcellulose at a concentration of 5 mg/mL) were administered to rats by intragastric gavage at a dose of 20 mg/kg (Ko et al. 2018). The control groups were administered with paeoniflorin (dissolved in normal saline containing 0.5% methylcellulose at a concentration of 5 mg/mL) by intragastric gavage at a dose of 20 mg/kg. Blood samples (250 μL) were collected into heparinized tubes via the *oculi chorioideae* vein at 0.083, 0.167, 0.33, 0.5, 1, 2, 4, 6, 8, 12, 24 and 36 h after the oral administration of paeoniflorin. The blood samples were centrifuged at 2200 *g* for 5 min, and the plasma samples obtained were stored at −40 °C until analysis.

### Instruments and conditions

The analysis was performed on an Agilent 1290 series liquid chromatography system and an Agilent 6460 triple-quadrupole mass spectrometer (Palo Alto, CA, USA) as previously reported (Liu et al. 2019). The chromatographic analysis of paeoniflorin was performed on a Waters X-Bridge C18 column (3.0 × 100 mm, i.d.; 3.5 μm, Milford, MA, USA) at room temperature (25 °C). The mobile phase was water (containing 0.1% formic acid) and acetonitrile (30:70, v: v) with isocratic elution at a flow rate of 0.3 mL/min, and the analysis time was 4 min. The injection volume was 2 μL, and the auto-sampler temperature was maintained at 25 °C.

The mass scan mode was the negative MRM mode. The precursor ion and product ion were *m/z* 524.8 → 449.0 for paeoniflorin and *m/z* 463.1 → 300.0 for hyperoside, respectively. The collision energy for puerarin and internal standard were 30 eV and 25 eV, respectively. The MS/MS conditions were optimized as follows: fragmentor, 120 V; capillary voltage, 4 kV; nozzle voltage, 500 V; nebulizer gas pressure (N_2_), 40 psig; drying gas flow (N_2_), 10 L/min; gas temperature, 350 °C; sheath gas temperature, 400 °C; and sheath gas flow, 11 L/min.

### Cell culture

The Caco-2 cell line was obtained from the American Type Culture Collection (Manassas, VA, USA). The Caco-2 cells were cultured in DMEM high glucose medium containing 15% FBS, 1% NEAA and 100 U/mL penicillin and streptomycin. The cells were cultured at 37 °C with 5% CO_2_. For transport studies, the cells at passage 40 were seeded on transwell polycarbonate insert filters (1.12 cm^2^ surface, 0.4 μm pore size, 12 mm diameter; Corning Costar Corporation, MA, USA) in 12-well plates at a density of 1 × 10^5^ cells/cm^2^. Cells were allowed to grow for 21 days. For the first seven days, the medium was replaced every two days, and then daily. The transepithelial electrical resistance (TEER) of the monolayer cells was measured using Millicell ERS-2 (Millipore Corporation, Billerica, MA, USA), and TEER exceeding 400 Ω·cm^2^ was used for the flux experiment. The integrity of the Caco-2 monolayers was confirmed by the paracellular flux of Lucifer yellow, which was less than 1% per hour. The alkaline phosphatase activity was validated using an Alkaline Phosphatase Assay Kit. The qualified monolayers were used for transport studies.

### Effects of glycyrrhizin on the transport of paeoniflorin in Caco-2 cell transwell model

Before the transport experiments, the cell monolayers were rinsed twice using warm (37 °C) Hanks’ balanced salt solution (HBSS), and then the Caco-2 cell transwell model were incubated at 37 °C for 20 min. After incubation, the Caco-2 cell transwell model were incubated with paeoniflorin in fresh incubation medium added on either the apical or basolateral side for the indicated times at 37 °C. The volume of incubation medium on the apical and basolateral sides was 0.5 mL and 1.5 mL, respectively, and a 100-μL aliquot of the incubation solution was withdrawn at the indicated time points from the receiver compartment and replaced with the same volume of fresh pre-warmed HBSS buffer. The efflux activity of *P-gp* was validated using a typical *P-gp* substrate digoxin (25 μM). The effects of glycyrrhizin or verapamil (*P-gp* inhibitor) on the transport of paeoniflorin were investigated by adding 50 μM glycyrrhizin or verapamil to both sides of the cell monolayers and pre-incubating the sample at 37 °C for 2 h. In addition, the effects of glycyrrhizin on the efflux of digoxin (25 μM) were also investigated. The permeability of paeoniflorin (10 μM) (which was validated for no obvious toxicity for Caco-2 cells within 2 h) in all of the above conditions for both directions, i.e., from the apical (AP) side to the basolateral (BL) side and from the BL side to the AP side, was measured after incubation for 30, 60, 90 and 120 min at 37 °C.

The apparent permeability coefficient (*P_app_*) was calculated using the equation of Artursson and Karlsson:

$$P_{\mathrm{app}}=\left(\Delta Q/\Delta t\right)\times [1/(A\times C_{0})]$$

where, *P_app_* is the apparent permeability coefficient (cm/s), ΔQ/Δt (μmol/s) is the rate at which the compound appears in the receiver chamber, C_0_ (μmol/L) is the initial concentration of the compound in the donor chamber and A (cm^2^) represents the surface area of the cell monolayer. Data were collected from three separate experiments, and each was performed in triplicate.

### Effects of glycyrrhizin on the metabolic stability of paeoniflorin in rat liver microsome incubation experiments

Rat liver microsomes were used to determine the phase I metabolic stability of paeoniflorin. The assay conditions and reaction mixtures were similar to those reported previously (Guo et al. 2018). In brief, except for NADPH-generating system (10 mM G-6-P, 1 mM NADP^+^, 4 mM magnesium chloride, 1 unit/mL of G-6-PDH), 10 μL rat liver microsomes (20 mg/mL), 4 μL paeoniflorin solution (100 μM) and 366 μL PBS buffer (0.1 M, *pH* 7.4) were added to the centrifuge tubes on ice. The reaction mixture was incubated at 37 °C for 5 min, and then NADPH-generating system (15 μL) was added. The effects of glycyrrhizin on the metabolic stability of paeoniflorin were investigated by adding 50 μM of glycyrrhizin to rat liver microsomes and pre-incubating them for 30 min at 37 °C, followed by the addition of paeoniflorin (100 μM). Aliquots of 30 μL were collected from reaction volumes at 0, 1, 3, 5, 15, 30 and 60 min, and 60-μL ice-cold acetonitrile containing IS was added to terminate the reaction, and then the concentration of paeoniflorin was determined by LC-MS/MS.

The *in vitro half-life* (*t_1/2_*) was obtained using the equation: *t_1/2_ = 0.693/k*; V (μL/mg) = volume of incubation (μL)/protein in the incubation (mg); Intrinsic clearance (Clint) (μL/min/mg protein) = v × 0.693/*t_1/2_*.

### Data analysis

The pharmacokinetic parameters, including the area under the plasma concentration-time curve (*AUC*), maximal plasma concentration (*C_max_*), the time for the maximal plasma concentration (*T_max_*) and the mean residence time (*MRT*), were calculated using the DAS 3.0 pharmacokinetic software (Chinese Pharmacological Association, Anhui, China).

The differences between the mean values were analyzed for significance using a one-way analysis of variance (ANOVA). Values of *p <* 0.05 were considered to be statistically significant.

## Results

### Effects of glycyrrhizin on the pharmacokinetics of paeoniflorin

**Figure 1.**
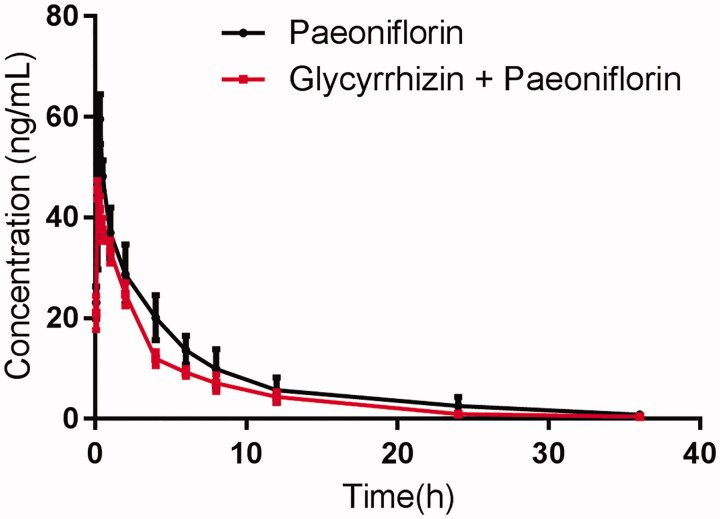
The pharmacokinetic profiles of paeoniflorin in male Sprague–Dawley rats after oral administration of 20 mg/kg paeoniflorin with or without glycyrrhizin (100 mg/kg/day for 7 days) pre-treatment. Each symbol with a bar represents the mean ± S.D. of six rats.

The mean plasma concentration-time curves of paeoniflorin after oral administration of paeoniflorin or oral administration of paeoniflorin with the pre-treatment of glycyrrhizin are presented in Figure 1. The pharmacokinetic parameters of paeoniflorin were calculated using the non-compartmental method with the DAS 3.0 pharmacokinetic software (Chinese Pharmacological Association, Anhui, China). The pharmacokinetic parameters are shown in Table 1.

**Table 1.** Pharmacokinetic parameter of paeoniflorin in rats after oral administration of paeoniflorin (20 mg/kg; *n* = 6, Mean ± S.D.) with or without treatment of glycyrrhizin.

Parameter	Control	Pre-treatment of glycyrrhizin
*T_max_* (h)	0.34 ± 0.02	0.17 ± 0.01*
*C_max_* (μg/L)	59.57 ± 10.24	45.36 ± 8.61*
*t*_1/2_ (h)	8.48 ± 2.01	5.88 ± 1.15*
*AUC_(0-inf)_* (μg h/L)	282.02 ± 35.06	202.29 ± 28.28*
*AUMC_(0-inf)_* (μg h/L)	2663.36 ± 287.25	1394.55 ± 227.62*
Oral CL (L/h/kg)	36.51 ± 5.28	49.75 ± 8.05*

* *p* < 0.05 indicate significant differences from the control.

As shown in Table 1, when the rats were pre-treated with glycyrrhizin (100 mg/kg) for 7 days, the *C_max_* decreased from 59.57 ± 10.24 to 45.36 ± 8.61 ng/mL, and the difference was significant (*p <* 0.05), and the *T_max_*, *AUC_(0-inf)_* and *AUMC_(0-inf)_* also decreased significantly (*p <* 0.05). These results indicated that glycyrrhizin could decrease the absorption and systemic exposure of paeoniflorin in rats when glycyrrhizin and paeoniflorin were co-administered. The *t_1/2_* value of paeoniflorin decreased from 8.48 ± 2.01 to 5.88 ± 1.15 h (*p <* 0.05), and oral clearance rate of paeoniflorin increased from 36.51 ± 5.28 to 49.75 ± 8.05 (*p <* 0.05), which indicated that the metabolism of paeoniflorin in rats was accelerated.

### Effects of glycyrrhizin on the bidirectional transport of paeoniflorin in Caco-2 cell transwell model

**Figure 2.**
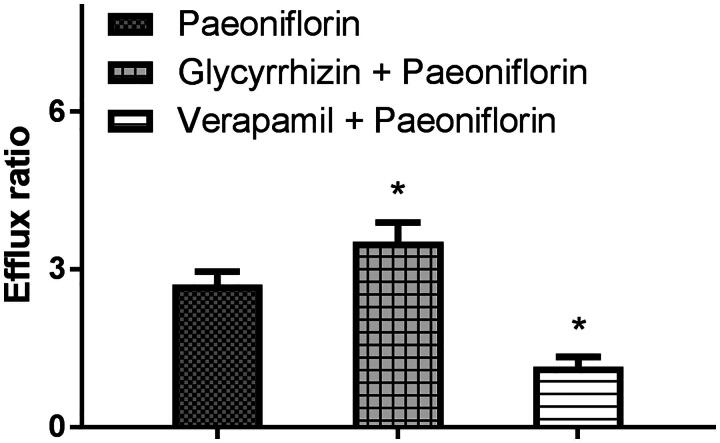
The effects of glycyrrhizin or verapamil on the efflux ratio of paeoniflorin in Caco-2 cell monolayer model. Caco-2 cell monolayers were incubated at 37 °C in HBSS (pH 7.4), and paeoniflorin (10 μM) was added to the donor chamber of the Caco-2 cell monolayer. Each symbol with a bar represents the mean ± S.D. of three repeated experiments.

To investigate the effects of *P-gp* on the transport of paeoniflorin, the Caco-2 cell transwell model was utilized. To validate the efflux activity of *P-gp*, a typical *P-gp* substrate digoxin was used, and the results indicated that the efflux ratio of digoxin was 11.57, which was abrogated in the presence of a typical *P-gp* inhibitor verapamil. As shown in Figure 2, after pre-treatment with glycyrrhizin for 2 h, the efflux ratio of digoxin increased from 12.20 to 15.36. The results indicated that the efflux activity of *P-gp* was qualified for the experiment. Then the transport of 10 μM of paeoniflorin across Caco-2 cell transwell model was investigated in this study. The *P_appAB_* and *P_appBA_* were 8.02 ± 0.57 × 10^−7 ^cm/s and 2.17 ± 0.37 × 10^−6^ cm/s, respectively. The *P_appBA_* was much higher than the *P_appAB_*, and the efflux ratio was 2.71, which indicated that efflux transporters might be involved in the transport of paeoniflorin. Then, the transport studies were performed in the presence of glycyrrhizin or verapamil to determine its effects on the transport of paeoniflorin. In the presence of 50 μM of glycyrrhizin, the *P_appAB_* decreased (7.15 ± 0.61 × 10^−7^ cm/s), whereas *P_appBA_* increased (2.52 ± 0.37 × 10^−6^ cm/s). The efflux ratio increased from 2.71 to 3.52. However, in the presence of verapamil (50 μM), a typical *P-gp* inhibitor, the efflux ratio decreased from 2.71 to 1.15. These results indicated that *P-gp* was involved in the transport of paeoniflorin in Caco-2 cell transwell model, and glycyrrhizin could enhance the efflux of paeoniflorin by inducing the activity of *P-gp*.

### Effects of glycyrrhizin on the metabolic stability of paeoniflorin in rat liver microsome incubation experiments

The effects of glycyrrhizin on the metabolic stability of paeoniflorin were investigated using rat liver microsomes. The metabolic half-life of paeoniflorin was 25.8 ± 3.7 min, and the intrinsic clearance rate of paeoniflorin was 53.7 ± 4.6 μL/min/mg protein, while in the presence of glycyrrhizin, the metabolic half-life (16.2 ± 3.1 min) was decreased, and the intrinsic clearance rate (85.6 ± 7.1 μL/min/mg protein) was increased. These results indicated that glycyrrhizin could accelerate the metabolism of paeoniflorin and increase the intrinsic clearance rate in rat liver.

## Discussion

The pharmacokinetic experiments showed that glycyrrhizin could decrease the absorption of paeoniflorin and increase the oral clearance rate of paeoniflorin in rats. Liu et al. (2019) have reported that verapamil, a typical *P-gp* and CYP3A4 inhibitor, could increase the systemic exposure of paeoniflorin when they were co-administered in rats, and these results indicated that CYP3A4 might be involved in the metabolism of paeoniflorin in rat liver. As some research articles have also indicated that glycyrrhizin could induce the activity of *P-gp* and CYP3A (Yan et al. 2017; Guo et al. 2018; Zhao et al. 2018), and therefore we speculated that glycyrrhizin might also decrease the absorption and accelerate the metabolism of paeoniflorin by enhancing *P-gp*-mediated drug efflux or CYP3A-mediated metabolism.

To investigate its potential mechanism, the Caco-2 cell transwell experiments were used, and the results indicated that *P-gp* was involved in the transport of paeoniflorin in Caco-2 cell transwell model, and glycyrrhizin could enhance the efflux of paeoniflorin by inducing the activity of *P-gp*.

The pharmacokinetic experiments also revealed that glycyrrhizin could decrease the *t_1/2_* value and increase the oral clearance rate of paeoniflorin, and these results were verified by using the rat liver microsome incubation experiments, as glycyrrhizin could increase the intrinsic clearance rate of paeoniflorin in rat liver.

Therefore, we suggest that when the rats were pre-treated with glycyrrhizin, the absorption of paeoniflorin would be decreased significantly, and the oral clearance rate of paeoniflorin would be increased. These results indicate that the drug–drug interaction between glycyrrhizin and paeoniflorin might appear when they were co-administered.

## Conclusions

This study suggests that co-administration of glycyrrhizin and paeoniflorin could decrease the systemic exposure of paeoniflorin. The *in vitro* Caco-2 cell transwell model and rat liver microsome incubation experiments showed glycyrrhizin might exert these effects through decreasing the absorption of paeoniflorin by inducing the activity of *P-gp* in intestine or through accelerating the metabolism of paeoniflorin by inducing the activity of CYP3A4 enzyme in rat liver. Clinically, combined medication of glycyrrhizin and paeoniflorin therapy would need dose increase to achieve comparable exposure of paeoniflorin.
